# Green Synthesis and Characterization of ZnO/CoFe_2_O_4_ Nanocomposites for Photocatalytic Degradation of Tetracycline Under Visible Light

**DOI:** 10.3390/molecules31162772

**Published:** 2026-08-09

**Authors:** Phan Thi Minh Huyen, Nguyen Xuan Dung

**Affiliations:** College of Education, Vinh University, Truong Vinh Ward, Nghe An 43131, Vietnam; huyenptm@vinhuni.edu.vn

**Keywords:** green synthesis, CoFe_2_O_4_ nanoparticles, ZnO nanoparticles, ZnO/CoFe_2_O_4_ nanocomposites, photocatalytic degradation, tetracycline

## Abstract

Antibiotic contamination of water, particularly by tetracycline (TC), requires effective and sustainable treatment strategies. In this study, CoFe_2_O_4_ nanoparticles were synthesized using lime juice as a natural stabilizing agent and combined with ZnO to obtain a ZnO/CoFe_2_O_4_ nanocomposite for visible-light-driven TC degradation. Complementary characterization confirmed the coexistence of ZnO and CoFe_2_O_4_ without detectable secondary phases, with predominantly spherical and irregular particles. The composite exhibited ferromagnetic behavior, suggesting potential magnetic recovery, and showed broader visible-light absorption and a reduced band gap of 3.05 eV compared with 3.23 eV for ZnO. Although its specific surface area and pore volume were lower than those of CoFe_2_O_4_, the nanocomposite displayed the highest photocatalytic performance. Under the optimized conditions of pH 6, 20 mg L^−1^ TC, and 1 g L^−1^ catalyst, 96.8% degradation was achieved after 120 min, with a pseudo-first-order rate constant of 0.029 min^−1^. The degradation efficiency remained 88.3% after five cycles. Scavenger experiments identified photogenerated holes (h^+^) and hydroxyl radicals (·OH) as the dominant reactive species. The improved photocatalytic activity may be associated with interfacial interactions between ZnO and CoFe_2_O_4_, suggesting that the green-synthesized nanocomposite has potential as a visible-light photocatalyst for TC degradation under the investigated conditions.

## 1. Introduction

Pharmaceutical contaminants are increasingly detected in aquatic environments and may cause persistent ecological and biological effects. Tetracycline (TC), widely used in human medicine and animal husbandry, is among the most common antibiotic pollutants, and its continuous discharge may promote antibiotic resistance and disrupt aquatic ecosystems [1].

Although conventional treatment methods can remove such contaminants, they may involve high costs, incomplete mineralization, or secondary waste. Photocatalytic advanced oxidation provides an alternative by generating reactive species capable of degrading persistent organic pollutants [2,3]. ZnO is inexpensive, abundant, and environmentally compatible but is limited by its wide bandgap, photocorrosion, and difficult recovery [4], whereas CoFe_2_O_4_ contributes magnetic responsiveness and visible-light absorption [5,6]. Combining ZnO with ferrite phases may improve light utilization and interfacial interactions while retaining magnetic recoverability [6,7,8]. Related studies also indicate that interfacial electron bridges, vacancy modulation, and confinement effects can influence photocatalytic performance [9,10].

Metal oxides such as ZnO and spinel ferrites such as CoFe_2_O_4_ have been synthesized through various physical and chemical routes, including precipitation, sol–gel processing, combustion, chemical vapor deposition, and laser ablation [11,12,13,14,15]. More sustainable strategies for producing photocatalytic materials have therefore attracted attention [16]. Bio-assisted synthesis using plant-derived compounds offers a more sustainable alternative [5,17,18]. Lime juice contains citric acid, ascorbic acid, sugars, and polyphenols that may contribute to metal-ion complexation and particle stabilization [18,19].

In this study, lime juice was used to assist the preparation of CoFe_2_O_4_ nanoparticles, which were subsequently combined with ZnO to form a ZnO/CoFe_2_O_4_ nanocomposite. The obtained materials were characterized, and their photocatalytic performance toward TC degradation under visible-light irradiation was evaluated.

## 2. Results and Discussion

### 2.1. Structural and Vibrational Characterization

**Figure 1:**
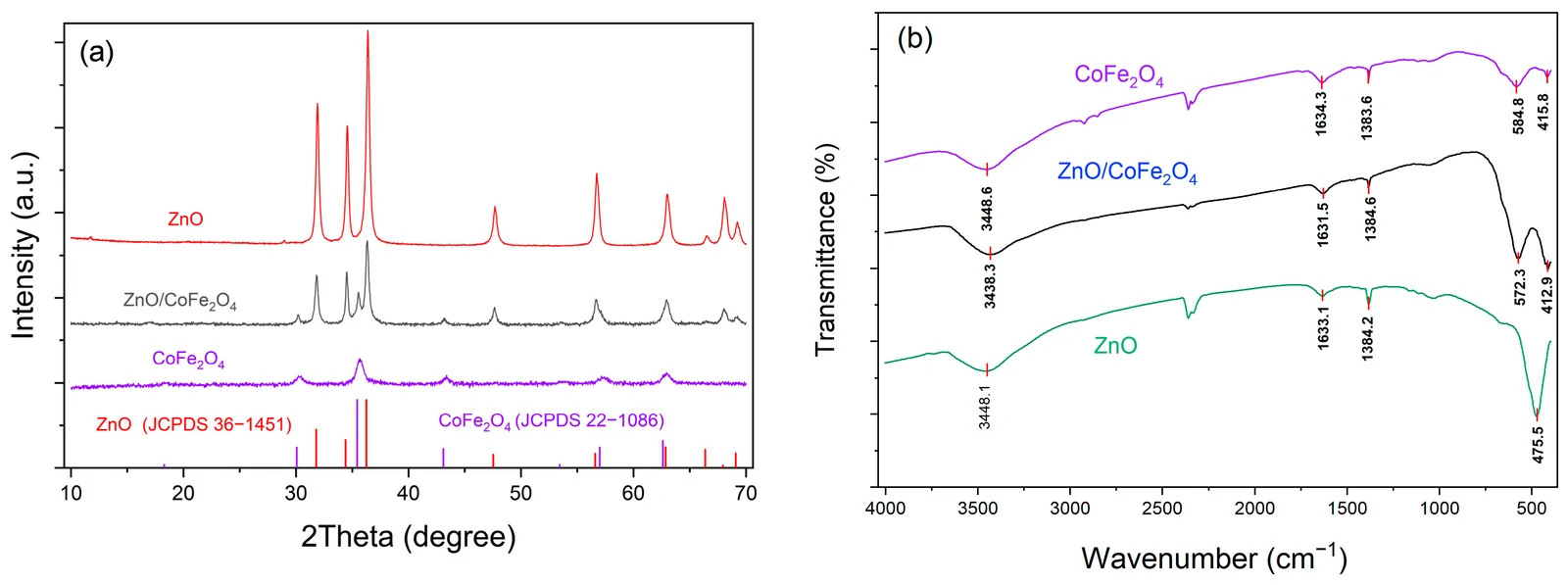
(**a**) X-ray diffraction patterns and (**b**) FTIR spectra of pristine ZnO, CoFe_2_O_4_, and the synthesized ZnO/CoFe_2_O_4_ nanocomposite.

Figure 1 presents the structural and vibrational characteristics of ZnO, CoFe_2_O_4_, and the ZnO/CoFe_2_O_4_ nanocomposite, as revealed by XRD patterns (Figure 1a) and FTIR spectra (Figure 1b). Figure 1a shows the XRD patterns of ZnO, CoFe_2_O_4_, and ZnO/CoFe_2_O_4_. Pure ZnO exhibited the characteristic reflections of hexagonal wurtzite ZnO (JCPDS No. 36-1451) at 2θ = 31.91°, 34.56°, 36.40°, 47.69°, 56.74°, 63.01°, 66.57°, 68.11°, and 69.29°, corresponding to the (100), (002), (101), (102), (110), (103), (200), (112), and (201) planes, respectively [20]. CoFe_2_O_4_ displayed the cubic spinel structure (JCPDS No. 22-1086), with peaks at 30.34°, 35.71°, 43.39°, 57.36°, and 63.01°, indexed to the (220), (311), (400), (511), and (440) planes [21]. The nanocomposite contained the characteristic reflections of both phases [6], with ZnO peaks at 31.86°, 34.51°, 36.33°, 47.64°, 56.66°, 66.51°, 68.10°, and 69.25° and CoFe_2_O_4_ peaks at 30.20°, 35.56°, 43.16°, and 62.95°. Their slight shifts toward lower 2θ values, accompanied by increased lattice parameters, may reflect lattice distortion and interfacial interactions, consistent with previous reports [14,22]. The reduced peak intensities may result from partial encapsulation or decreased crystallinity [7,23,24], while peak overlap occurred at 35.5–36.5°. No additional reflections were observed, indicating the absence of detectable secondary iron- or cobalt-oxide phases.

The crystallite sizes and crystallographic parameters were calculated using the Scherrer equation and the standard relationships for hexagonal ZnO and cubic CoFe_2_O_4_ [25,26,27]. The corresponding calculation procedures, equations, and complete data are provided in Section S4 and Table S1. The crystallite sizes of the ZnO and CoFe_2_O_4_ phases increased from 15.90 and 11.03 nm in the pristine materials to 21.67 and 12.84 nm in the nanocomposite, respectively. Slight increases in the lattice parameters and unit-cell volumes, accompanied by slight decreases in the X-ray densities, may reflect lattice expansion and interfacial strain. These trends are consistent with the observed peak shifts toward lower 2θ values and previous reports [14,15,23].

Complementary information on the bonding characteristics was obtained from the FTIR spectra shown in Figure 1b. All samples exhibited broad bands at approximately 3438–3449 and 1631–1634 cm^−1^, assigned to O–H stretching and bending vibrations of surface hydroxyl groups and adsorbed water, respectively [28,29,30]. The weak bands near 1384 cm^−1^ were attributed to residual organic species from the synthesis process [6,26,31,32]. ZnO exhibited a characteristic Zn–O vibration at 475.5 cm^−1^, whereas CoFe_2_O_4_ showed metal–oxygen vibrations at 584.8 and 415.8 cm^−1^, corresponding to the tetrahedral and octahedral sites of the spinel structure, respectively [33,34]. In the composite, the corresponding ferrite bands occurred at 572.3 and 412.9 cm^−1^, while the Zn–O contribution was not resolved separately because of band overlap in the low-wavenumber region. The downshift of the high-frequency M–O band may reflect a change in the local bonding environment upon composite formation [27,33,34].

### 2.2. Morphological and Elemental Analysis

#### 2.2.1. SEM and TEM Characterization

**Figure 2:**
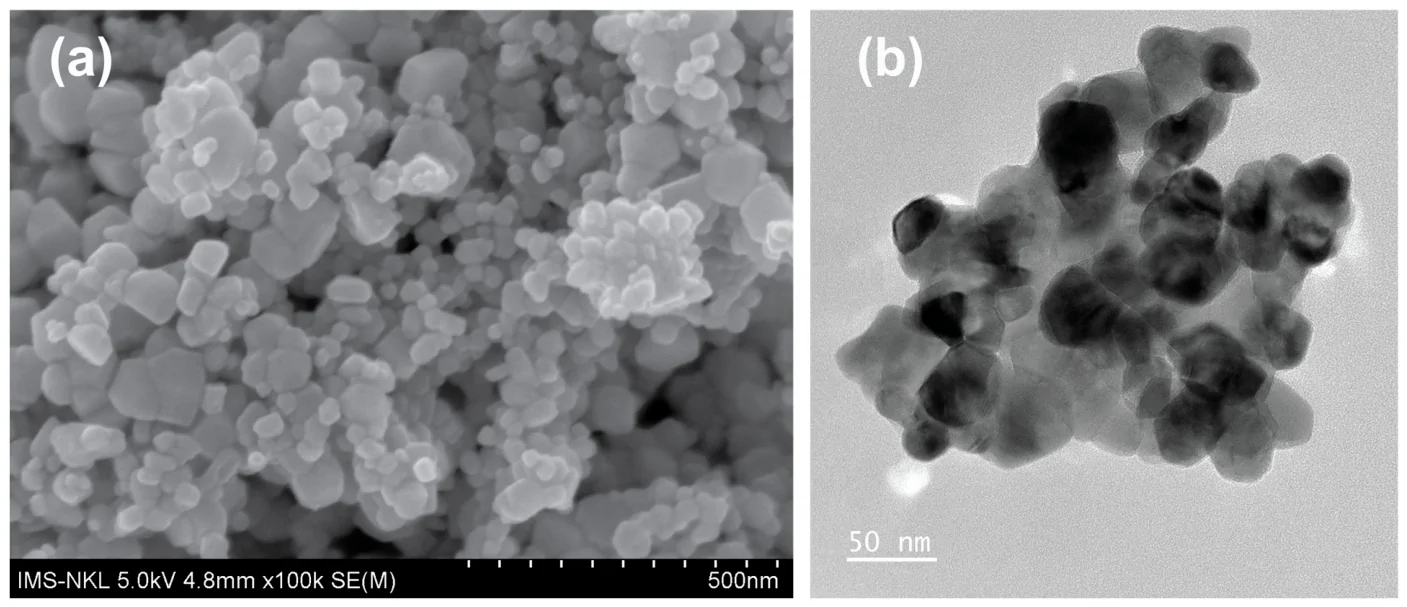
(**a**) FE-SEM and (**b**) TEM images of the ZnO/CoFe2O4 nanocomposite.

FE-SEM and TEM were used to examine the morphology of ZnO, CoFe_2_O_4_, and the ZnO/CoFe_2_O_4_ nanocomposite. Images of the pristine materials are provided in Figure S1. ZnO exhibited predominantly spherical-to-quasi-spherical particles with sizes of approximately 10–20 nm (Figure S1a,b), whereas CoFe_2_O_4_ showed smaller, more strongly agglomerated particles with less distinct grain boundaries (Figure S1c,d). This aggregation may be associated with the magnetic nature and higher surface area of CoFe_2_O_4_ [23,35,36,37]. As shown in Figure 2a,b, the ZnO/CoFe_2_O_4_ nanocomposite consisted of approximately spherical and irregularly shaped particles ranging from 10 to 30 nm, together with some larger agglomerates. The comparatively smoother and more defined particle surfaces may result from ZnO deposition on magnetic CoFe_2_O_4_ particles [36]. TEM observations also revealed relatively larger particles and contrast variations in the nanocomposite compared with pristine CoFe_2_O_4_. These features may promote close interfacial contact between the two phases and thereby contribute to the photocatalytic performance of the ZnO/CoFe_2_O_4_ nanocomposite.

#### 2.2.2. EDX Analysis

Energy-dispersive X-ray (EDX) analysis was performed to verify the elemental composition and purity of pure ZnO, pure CoFe_2_O_4_, and the ZnO/CoFe_2_O_4_ nanocomposite, as shown in Figure S2a, S2b and S2c, respectively. The EDX spectra of pure ZnO show Zn and O signals, while pure CoFe_2_O_4_ exhibits characteristic peaks corresponding to Co, Fe, and O, confirming the successful formation of each individual phase. For the nanocomposite, the simultaneous presence of Zn, Co, Fe, and O confirms the integration of both components [14]. Additionally, Table 1 summarizes the weight and atomic percentages obtained experimentally from EDX analysis and theoretically calculated from the chemical formula. The good agreement between the theoretical and experimental compositions further confirms the expected stoichiometry of the synthesized materials.

### 2.3. UV–Visible Diffuse Reflectance Spectroscopy (DRS)

**Figure 3:**
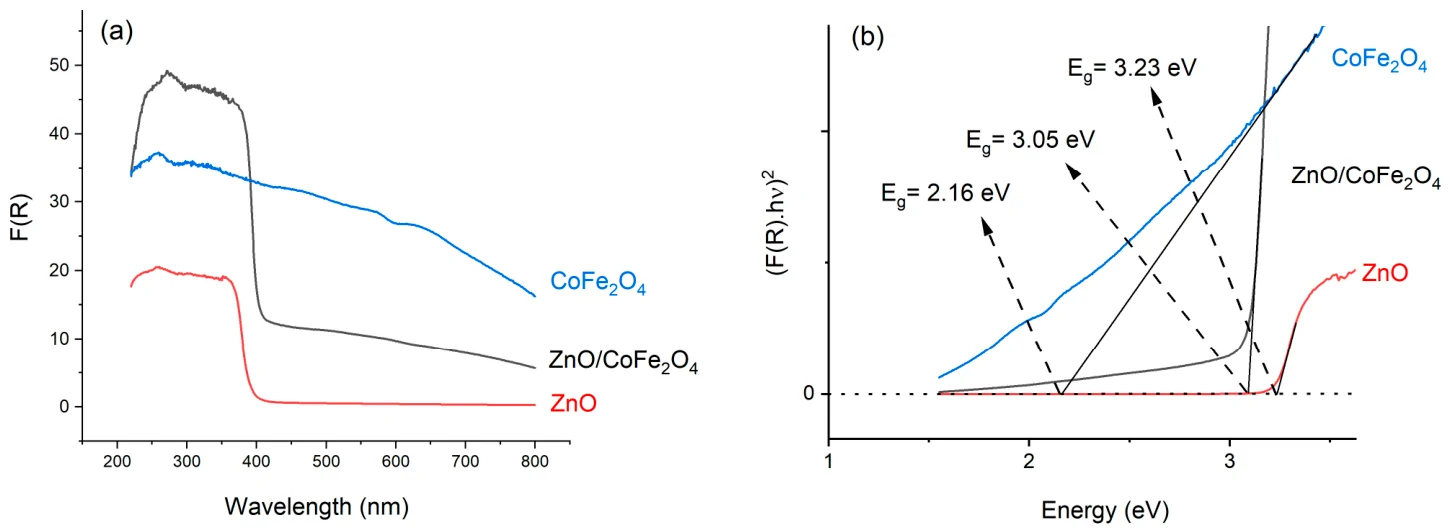
(**a**) Kubelka–Munk-transformed UV–Vis diffuse reflectance spectra of ZnO, CoFe_2_O_4_, and ZnO/CoFe_2_O_4_; and (**b**) the corresponding Tauc plots used to estimate the optical band-gap energies. The solid lines in (**b**) represent the linear extrapolations used to determine the band-gap energies ($E_{g}$).

The optical properties and light-harvesting characteristics of ZnO, CoFe_2_O_4_, and the ZnO/CoFe_2_O_4_ nanocomposite were investigated by UV–Vis diffuse reflectance spectroscopy (DRS), as shown in Figure 3. Because the samples were solid powders, the reflectance data were transformed using the Kubelka–Munk function, $F(R)=(1-R{)}^{2}/2R$, where R is the diffuse reflectance. As shown in Figure 3a, pure ZnO exhibits a pronounced optical response mainly in the ultraviolet region, with a sharp absorption edge at approximately 380–400 nm, corresponding to the intrinsic transition from the O 2p valence band to the Zn 4s conduction band [38]. In contrast, magnetic CoFe_2_O_4_ NPs possess absorption extending significantly into the visible light region, a characteristic property of many iron-based oxides that enhances their solar energy utilization [39]. For the nanocomposite, the Kubelka–Munk spectrum exhibits a characteristic red shift and broadening of the absorption edge into the visible range compared to pristine ZnO [24]. The Tauc method was employed to determine the optical band-gap energy ($E_{g}$) by plotting ${\left(F(R)h\nu \right)}^{2}$ versus photon energy (hν) and extrapolating only the linear portion of the fundamental absorption edge to the energy axis, consistent with a direct allowed transition for these materials. Based on the Tauc plots (Figure 3b), the $E_{g}$ values of pure ZnO, pure CoFe_2_O_4_, and the ZnO/CoFe_2_O_4_ nanocomposite were estimated to be 3.23, 2.16, and 3.05 eV, respectively. The calculated value for the composite was reduced relative to that of bare ZnO, a phenomenon that may be associated with interactions between ZnO and CoFe_2_O_4_ in the composite [23,40]. This narrowed bandgap suggests an enhanced ability of the composite to harvest visible light; however, it does not directly demonstrate improved charge-carrier separation [40,41].

### 2.4. XPS Analysis

**Figure 4:**
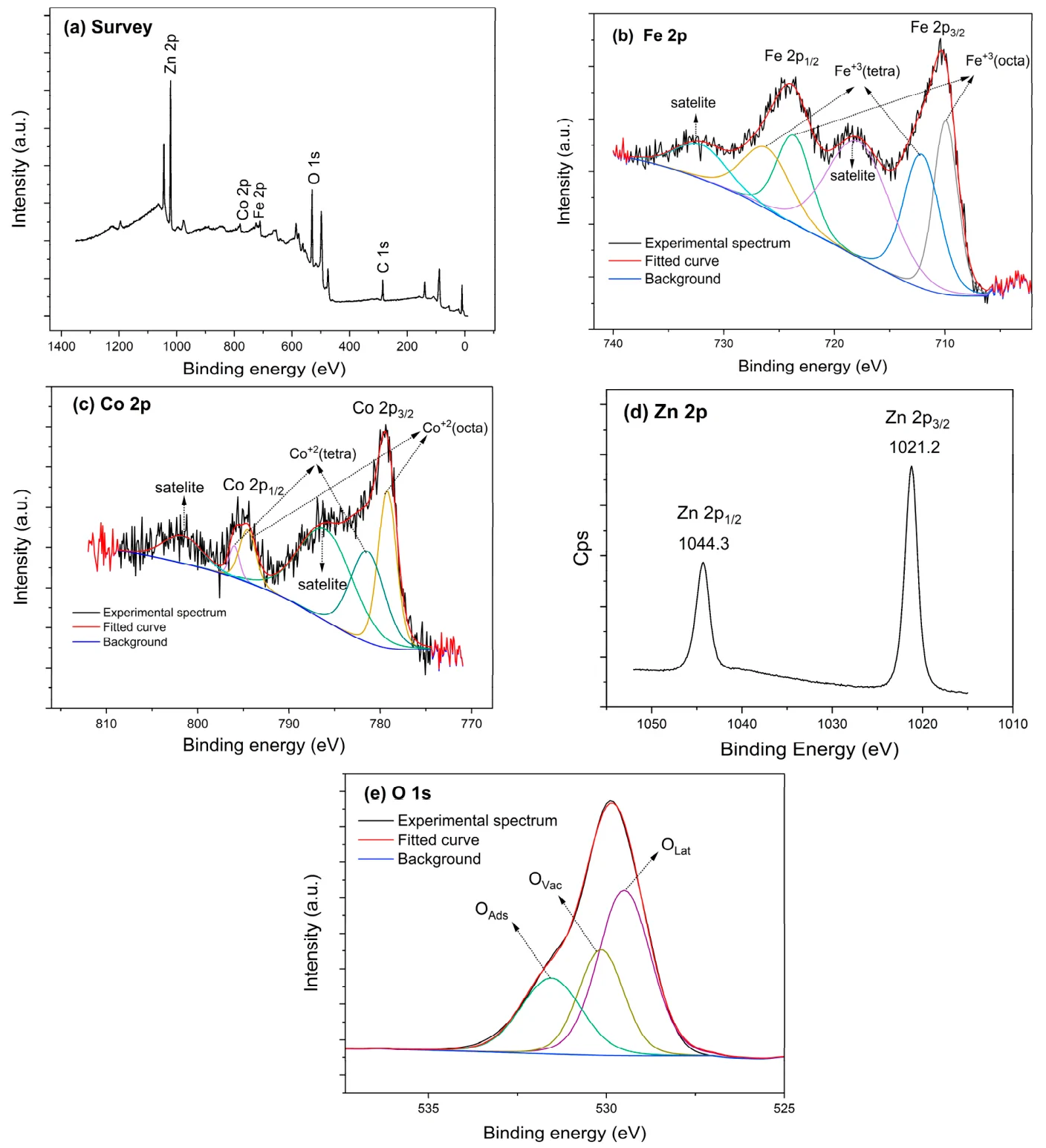
XPS spectra of ZnO/CoFe_2_O_4_: (**a**) XPS survey spectra, (**b**) Fe 2p and (**c**) Co 2p spectra, (**d**) Zn 2p, and (**e**) O 1s.

The XPS results displayed in Figure 4 provide information on the surface elemental composition and chemical valence states of the ZnO/CoFe_2_O_4_ nanocomposite. As shown in Figure 4a, the survey XPS spectrum of the ZnO/CoFe_2_O_4_ nanocomposite displays characteristic peaks of Zn, Co, Fe, and O, confirming the successful formation of the nanocomposite with the expected elemental composition. In addition, a weak carbon signal was detected, which is generally attributed to adventitious carbon species adsorbed on the sample surface during handling [14,27]. Figure 4b–e illustrate the high-resolution XPS spectra corresponding to Fe 2p, Co 2p, Zn 2p, and O 1s, respectively. To gain further insight into the oxidation states and site occupancy of the metal ions, the Fe 2p and Co 2p spectra were deconvolved into their individual components corresponding to the tetrahedral (A) and octahedral (B) positions within the spinel structure. The high-resolution Fe 2p spectrum (Figure 4b) reveals two distinct spin–orbit doublets, with satellite peaks at 717.8 and 732.3 eV. The peaks at 710.0 eV (Fe 2p_3/2_) and 723.7 eV (Fe 2p_1/2_) are assigned to Fe^3+^ ions at octahedral (B) sites, while those at 712.1 eV (Fe 2p_3/2_) and 726.5 eV (Fe 2p_1/2_) correspond to Fe^3+^ ions occupying tetrahedral (A) sites in the spinel structure of CoFe_2_O_4_ [14,42,43]. The high-resolution Co 2p spectrum (Figure 4c) shows the presence of Co^2+^ spin−orbit doublets: Co 2p_3/2_ (779.3 for Co^2+^ in B sites and 781.5 eV for Co^2+^ in A sites) and Co 2p_1/2_ (794.5 eV for Co^2+^ in B sites and 796.0 eV for Co^2+^ in A sites). Corresponding satellite peaks were observed at 786.0 and 801.7 eV [14]. No evidence of Co^3+^ species was detected in the XPS spectrum [44]. Moreover, the spin–orbit splitting of approximately 16 eV between the Co 2p_3/2_ and Co 2p_1/2_ components, together with the observed satellite peaks, is consistent with the presence of Co^2+^ ions in the spinel CoFe_2_O_4_ lattice [42,45].

As illustrated in Figure 4d, the high-resolution Zn 2p spectrum consists of two well-defined peaks located at 1021.2 and 1044.3 eV, corresponding to Zn 2p_3/2_ and Zn 2p_1/2_, respectively. The spin–orbit splitting energy between these two components was determined to be approximately 23.1 eV, which is characteristic of Zn^2+^ species in ZnO. These binding energy values agree well with those reported in the literature, confirming the presence of ZnO and its successful incorporation into the ZnO/CoFe_2_O_4_ nanocomposite [46].

As displayed in Figure 4e, the O 1s spectrum of ZnO/CoFe_2_O_4_ was deconvolved into three distinct components centered at binding energies of 529.5, 530.2, and 532.1 eV. The peak at 529.5 eV (denoted as O_Lat_) corresponds to lattice oxygen species within Co–O and Fe–O bonds, whereas the feature at 530.2 eV (O_Vac_) is associated with oxygen vacancies. The highest-energy component at 532.1 eV (O_Ads_) arises from hydroxyl groups chemisorbed onto the surface of the catalyst [47].

### 2.5. BET Analysis and Magnetic Properties

**Figure 5:**
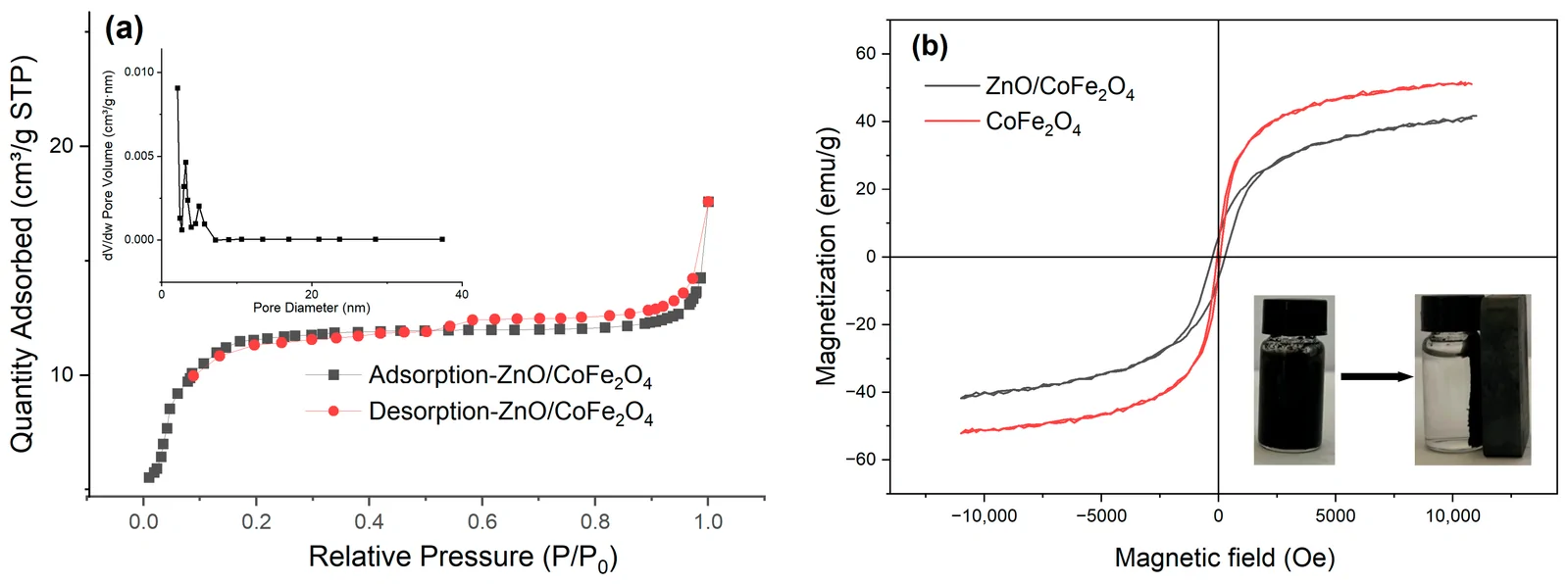
(**a**) N_2_ adsorption–desorption isotherms and corresponding BJH pore-size distributions of ZnO/CoFe_2_O_4_, and (**b**) room-temperature magnetization curves of CoFe_2_O_4_ and ZnO/CoFe_2_O_4_.

Nitrogen adsorption–desorption analysis was further conducted to determine the specific surface area, pore diameter, and pore volume of ZnO, CoFe_2_O_4_, and ZnO/CoFe_2_O_4_. The adsorption–desorption isotherms and corresponding BJH pore-size distributions of pristine ZnO and CoFe_2_O_4_ are provided in Figure S3a,b, whereas those of ZnO/CoFe_2_O_4_ are shown in Figure 5a. The specific surface areas, total pore volumes, and average pore diameters of the three materials are summarized in Table S2.

Pure ZnO exhibited a relatively low BET specific surface area of 10.9 m^2^ g^−1^, with a pore volume of 0.011 cm^3^ g^−1^ and an average pore diameter of 15.7 nm. Based on the IUPAC classification, the N_2_ adsorption–desorption isotherm can be identified as a type II isotherm with an H4 hysteresis loop in the high relative pressure ($P/P_{0}$) range of 0.84–0.98, indicating the mesoporous nature of ZnO NPs’ narrow and irregular slit-like pore shapes with a wide diameter distribution and walls formed of ordered mesoporous materials [48]. In contrast, pure CoFe_2_O_4_ exhibited a significantly higher BET surface area of 75.3 m^2^ g^−1^ and a larger pore volume of 0.157 cm^3^ g^−1^ compared with pure ZnO, while its average pore diameter decreased to 11.1 nm. Its N_2_ adsorption–desorption isotherm was classified as type IV with an H3 hysteresis loop, suggesting a more developed mesoporous structure associated with slit-shaped pores and interparticle voids generated by ferrite nanoparticle aggregation [49].

For the ZnO/CoFe_2_O_4_ composite, the BET surface area decreased to 36.8 m^2^ g^−1^ and the pore volume dropped to 0.022 cm^3^ g^−1^ compared with pure CoFe_2_O_4_. The decrease in the mass-normalized BET surface area of the nanocomposite is also consistent with the substantially lower surface area of ZnO (10.9 m^2^ g^−1^) than that of CoFe_2_O_4_ (75.3 m^2^ g^−1^). In addition, ZnO deposition and particle aggregation may partially cover accessible surface sites and block interparticle voids or pore entrances, thereby reducing the accessible surface area [36,40]. Meanwhile, the average pore diameter further decreased to 3.5 nm, and the composite retained a type IV isotherm with an H3 hysteresis loop, confirming the formation of smaller mesopores where adsorption occurs through multilayer adsorption followed by capillary condensation [36,50]. These results confirm that the integration of ZnO with CoFe_2_O_4_ significantly modifies the textural properties and pore structure of the composite material.

Figure 5b presents the room-temperature magnetization curves of CoFe_2_O_4_ NPs and the ZnO/CoFe_2_O_4_ nanocomposite. The magnetization curve of CoFe_2_O_4_ NPs shows ferromagnetic behavior with a saturation magnetization ($M_{s}$) of 52.2 emu g^–1^, a remanent magnetization (M_r_) of 3.9 emu g^–1^, and a coercivity (H_c_) of 71.4 Oe. Meanwhile, the nanocomposite presents a saturation magnetization of 41.6 emu g^–1^, a remanent magnetization of 5.9 emu g^–1^, and a coercivity of 296.8 Oe. These results reveal a noticeable decrease in the saturation magnetization of the ZnO/CoFe_2_O_4_ nanocomposite compared with pristine CoFe_2_O_4_ NPs, which is mainly attributed to the incorporation of non-magnetic ZnO into the composite structure [23,51]. In contrast, a slight increase in $M_{s}$ after ZnO incorporation was reported and attributed to ferromagnetic coupling between the ferrimagnetic CoFe_2_O_4_ phase and ZnO nanoparticles [14].

As shown in the inset of Figure 5b, the ZnO/CoFe_2_O_4_ nanocomposite can be effectively collected under an external magnetic field owing to the presence of the CoFe_2_O_4_ component. The observed magnetic responsiveness suggests that the catalyst may potentially be separated from aqueous media using an external magnetic field, which is advantageous for practical photocatalytic applications and catalyst recyclability.

### 2.6. Photocatalytic Degradation

#### 2.6.1. TC Removal Efficiency for ZnO, CoFe_2_O_4_, and ZnO/CoFe_2_O_4_

**Figure 6:**
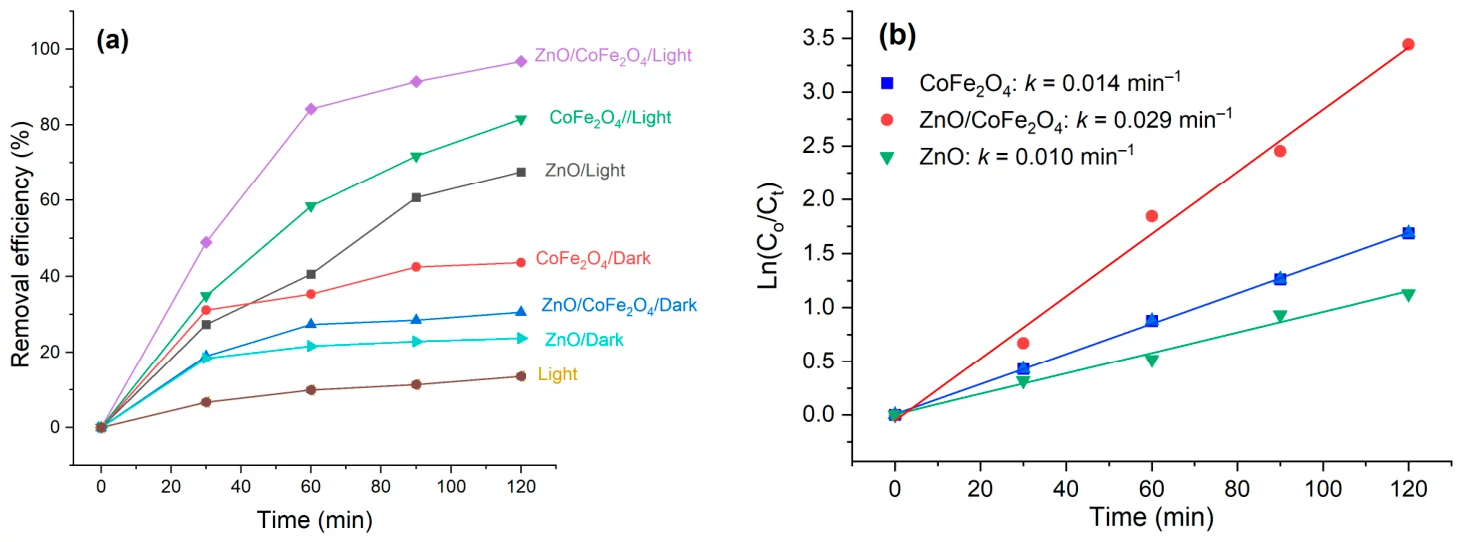
(**a**) Photocatalytic degradation of tetracycline (TC) over ZnO, CoFe_2_O_4_, and ZnO/CoFe_2_O_4_, (**b**) pseudo-first-order kinetic plots for ZnO, CoFe_2_O_4_, and ZnO/CoFe_2_O_4_.

Figure 6 compares the tetracycline removal performance of ZnO, CoFe_2_O_4_, and ZnO/CoFe_2_O_4_ under both dark and illuminated conditions. Under dark-control conditions, the removal efficiencies achieved by ZnO, CoFe_2_O_4_, and ZnO/CoFe_2_O_4_ were approximately 24%, 44%, and 31%, respectively, reflecting the adsorption of TC molecules onto the catalyst surfaces. Among the investigated materials, CoFe_2_O_4_ exhibited the highest adsorption capacity, which can be attributed to its larger specific surface area and the resulting increase in available adsorption sites. In contrast, direct photolysis of TC in the absence of any catalyst resulted in only about 13% removal, indicating that light irradiation alone has a limited effect on TC degradation.

After 120 min of light irradiation, the removal efficiencies of TC over ZnO, CoFe_2_O_4,_ and ZnO/CoFe_2_O_4_ were 67.6%, 81.5% and 96.8%, respectively (Figure 6a). Furthermore, the pseudo-first-order kinetic rate constants (*k*) of ZnO, CoFe_2_O_4_, and ZnO/CoFe_2_O_4_ were determined to be 0.010, 0.014, and 0.029 min^−1^, respectively (Figure 6b). The significantly higher reaction rate of the ZnO/CoFe_2_O_4_ nanocomposite suggests a synergistic effect between ZnO and CoFe_2_O_4_ in enhancing photocatalytic activity [36,52]. This improvement may be associated with interfacial interactions between ZnO and CoFe_2_O_4_, which could promote charge separation and interfacial electron transfer, suppress the recombination of photogenerated electron–hole pairs, and prolong charge carrier lifetime, thereby facilitating the generation of reactive oxygen species (ROS), particularly hydroxyl radicals (·OH), for improved TC degradation [23].

#### 2.6.2. Effects of Operating Parameters on TC Photocatalytic Degradation

**Figure 7:**
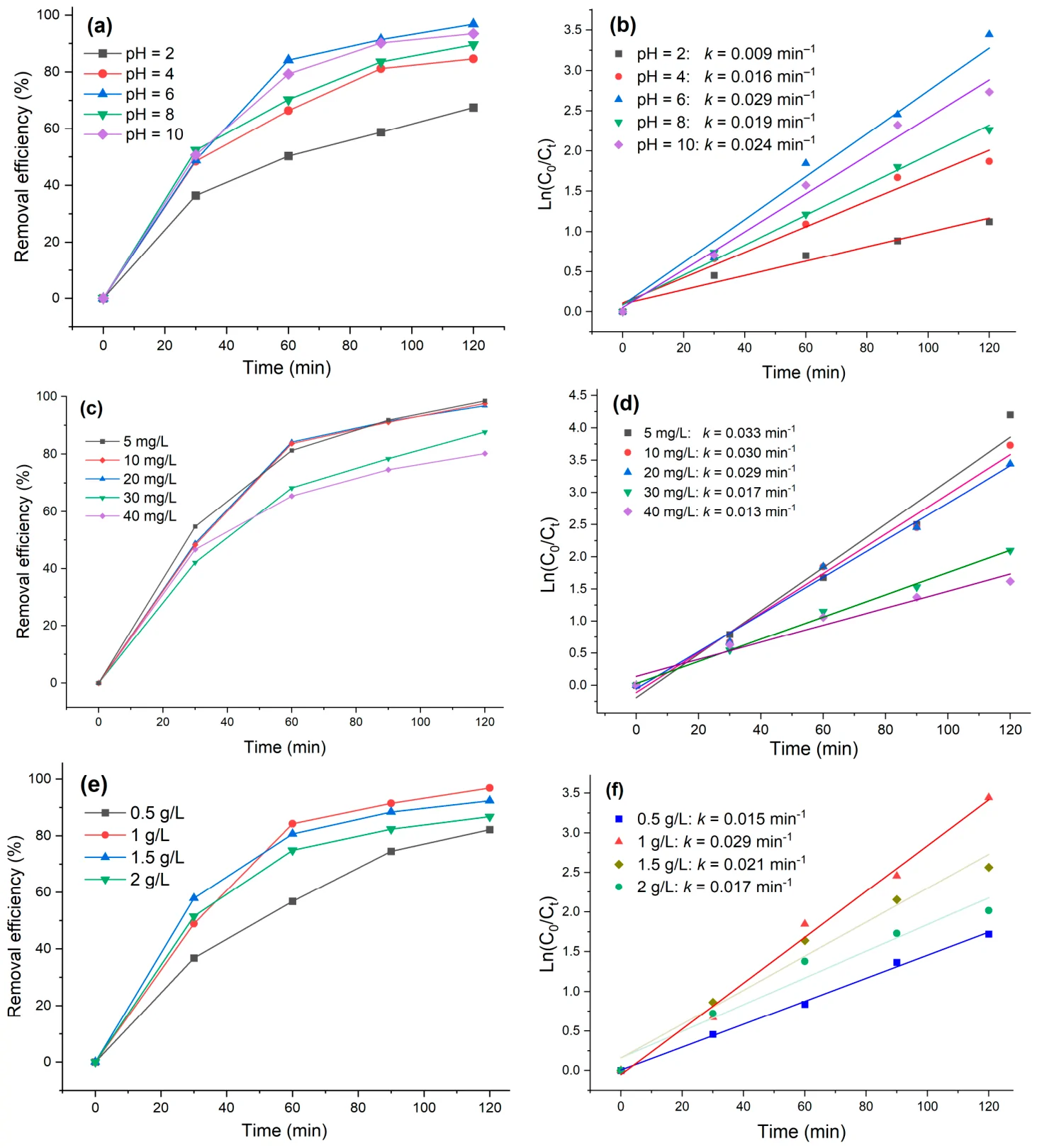
Effects of operating parameters on TC photocatalytic degradation over the ZnO/CoFe_2_O_4_ nanocomposite: (**a**) effect of initial solution pH on TC removal efficiency; (**b**) corresponding pseudo-first-order kinetic plots at different pH values; (**c**) effect of initial TC concentration on removal efficiency; (**d**) corresponding pseudo-first-order kinetic plots at different initial TC concentrations; (**e**) effect of photocatalyst dosage on removal efficiency; and (**f**) corresponding pseudo-first-order kinetic plots at different photocatalyst dosages.

The effects of initial solution pH, initial TC concentration, and photocatalyst dosage on the photocatalytic degradation of TC over the ZnO/CoFe_2_O_4_ nanocomposite were investigated. The corresponding TC removal efficiencies and pseudo-first-order kinetic plots are summarized in Figure 7a–f.

Effect of Initial Solution pH. The pH of the aqueous medium significantly affects the photocatalytic degradation process by influencing both the catalyst surface properties and pollutant speciation [53]. The pH of the solution affects the surface charge of photocatalysts, the charge of pollutant species, and the surface adsorption of pollutant species. TC exhibits amphoteric behavior with three dissociation constants (pKa = 3.30, 7.68, and 9.70), resulting in different ionic forms depending on the solution pH. The dominant species are H_4_TC^+^ at pH < 3.4, H_3_TC at pH 3.4–7.6, H_2_TC^−^ at pH 7.6–9.7, and HTC^2−^ at pH > 9.7 [54,55]. The point of zero charge (pH_PZC_) is a fundamental surface property of solid particles in aqueous media, representing the specific pH level at which the net electrical charge on the particle surface is zero [56]. As shown in Figure S4, the ${pH}_{PZC}$ of the ZnO/CoFe_2_O_4_ nanocomposite was determined to be 6.8. Therefore, the composite surface carries a positive charge below this pH and a negative charge above it.

Figure 7a,b clearly demonstrate that the TC removal efficiency increased significantly from 67.5% at pH 2 to 96.8% at pH 6, then decreased to 89.6% at pH 8 before slightly increasing to 93.5% at pH 10. At pH 2, both ZnO/CoFe_2_O_4_ and TC molecules are positively charged, resulting in strong electrostatic repulsion and weak adsorption, which limits the photocatalytic degradation process [52]. At pH 4 and 6, TC predominantly exists in its neutral form, while the catalyst surface remains positively charged, resulting in stronger interactions between TC molecules and the catalyst surface [39]. However, at pH 8, TC predominantly exists as H_2_TC^−^ species, while the catalyst surface becomes negatively charged, causing electrostatic repulsion and a slight decrease in degradation efficiency. The increased TC degradation efficiency at pH 10, despite the stronger electrostatic repulsion between the negatively charged catalyst surface and dianionic (HTC^2−^), can be attributed to the abundant $OH^{-}$ ions in alkaline conditions, which promote the generation of more highly reactive ·OH radicals. In addition, TC becomes less stable at a high pH and is more susceptible to photodegradation, thereby enhancing its removal efficiency [57].

This behavior was consistent with the pseudo-first-order kinetic analysis shown in Figure 7b. The rate constant increased from 0.009 min^−1^ at pH 2 to 0.029 min^−1^ at pH 6, indicating enhanced photocatalytic activity under near-neutral conditions. The k value then decreased to 0.019 min^−1^ at pH 8 due to electrostatic repulsion between negatively charged TC species and the catalyst surface, before increasing again to 0.024 min^−1^ at pH 10 as a result of increased ·OH radical generation in alkaline conditions. Based on both the degradation efficiency and pseudo-first-order kinetic results, pH 6 was considered the optimal condition for TC photocatalytic degradation over the ZnO/CoFe_2_O_4_ photocatalyst due to its highest reaction rate and excellent removal performance.

Effect of Initial TC Concentration. The influence of the initial TC concentration on photocatalytic degradation was evaluated over the concentration range of 5–40 mg/L (Figure 7c). The ZnO/CoFe_2_O_4_ photocatalyst exhibited excellent performance at low TC concentrations, achieving a maximum degradation efficiency of 98.5% at 5 mg/L. The removal efficiency remained high at 97.6% and 96.8% for TC concentrations of 10 and 20 mg/L, respectively. However, further increasing the TC concentration to 30 and 40 mg/L resulted in a marked decrease in degradation efficiency, reaching 87.7% and 80.2%, respectively. This reduction can be attributed to greater initial surface coverage and stronger competition among TC molecules for the available adsorption and reaction sites at higher pollutant concentrations. In addition, excessive TC molecules may hinder light penetration and compete for photogenerated reactive species, thereby reducing the overall photocatalytic degradation efficiency [58,59,60].

Figure 7d presents the pseudo-first-order kinetic analysis for TC photodegradation at different initial concentrations. The calculated rate constants (k) for TC concentrations of 5, 10, 20, 30, and 40 mg/L were 0.033, 0.030, 0.029, 0.017, and 0.013 min^−1^, respectively. A gradual decrease in the reaction rate was observed with increasing TC concentration, indicating that higher pollutant concentrations negatively affect the photocatalytic process. This behavior can be attributed to greater initial surface coverage, reduced light penetration, and increased competition among TC molecules for reactive species, particularly during the early and intermediate stages of irradiation. An initial concentration of 20 mg/L was selected as the reference working concentration for subsequent experiments, providing a balance between reaction kinetics and pollutant loading under the investigated laboratory conditions. While lower concentrations typically yield higher pseudo-first-order rate constants due to an abundance of reactive species relative to pollutant molecules, the selection of 20 mg/L provides a suitable balance between high removal efficiency and a measurable pollutant concentration [54,61,62]. Furthermore, at this concentration, the ‘inner filter effect’ and excessive initial surface coverage are less pronounced than those at higher levels (>20 mg/L), which can impede photon penetration and reactive-species generation, thereby maintaining high degradation efficiency under the controlled experimental conditions [60,61,63].

Effect of Photocatalyst Dosage. Previous studies have demonstrated that determining an optimal catalyst dosage is crucial for achieving maximum photocatalytic degradation efficiency of pollutants such as TC, and this optimum value depends on the physicochemical properties and composition of the photocatalyst [63,64]. The effect of photocatalyst dosage on TC degradation was evaluated by varying the concentration of the ZnO/CoFe_2_O_4_ nanocomposite from 0.5 to 2.0 g/L. The corresponding TC removal efficiencies and pseudo-first-order kinetic plots are presented in Figure 7e,f, respectively.

As shown in Figure 7e,f, increasing the ZnO/CoFe_2_O_4_ dosage from 0.5 to 1.0 g/L significantly improved TC degradation, with the removal efficiency rising from 82.1% to 96.8% and the rate constant increasing from 0.015 to 0.029 min^−1^. This enhancement can be attributed to the greater availability of active sites and larger surface area, which facilitate TC adsorption, light absorption, and reactive oxygen species (ROS) generation [63,65]. In contrast, further increasing the catalyst dosage beyond 1.0 g/L resulted in a gradual decline in photocatalytic performance. At catalyst loadings of 1.5 and 2.0 g/L, the degradation efficiencies decreased to 92.3% and 86.7%, while the corresponding rate constants dropped to 0.021 and 0.017 min^−1^, respectively. This reduction is likely caused by particle agglomeration and increased suspension turbidity, which diminishes the effective surface area and restricts light penetration, thereby suppressing ROS production [63,66]. Consequently, a catalyst dosage of 1.0 g/L was determined to be optimal for TC degradation under the investigated conditions.

#### 2.6.3. The Recyclability Evaluation and Photocatalytic Mechanism

**Figure 8:**
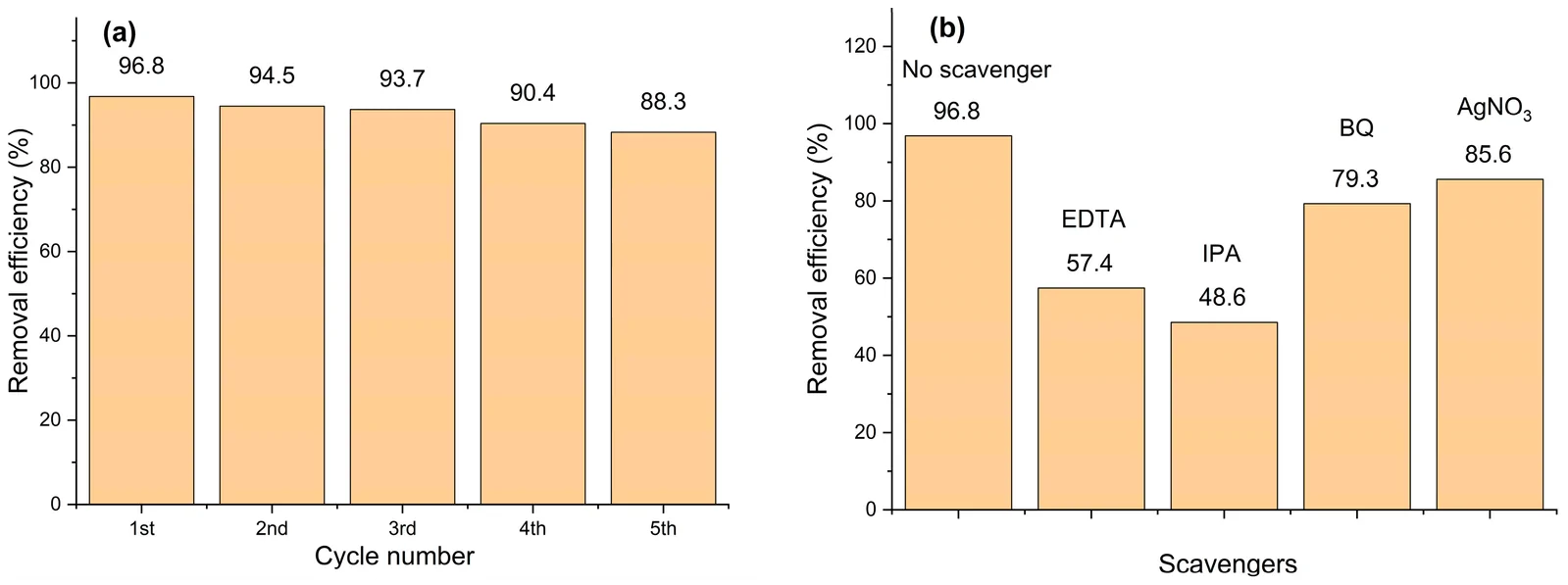
(**a**) Reusability of the ZnO/CoFe_2_O_4_ nanocomposite for photocatalytic degradation of TC. (**b**) The effect of scavengers (EDTA, IPA, BQ, and AgNO_3_) on the removal efficiency of TC.

Catalytic stability is an important factor in evaluating photocatalytic performance and reducing the long-term operational cost for organic pollutant treatment. To investigate the stability and reusability of the ZnO/CoFe_2_O_4_ nanocomposite, recycling experiments were carried out under the optimized conditions, including pH 6, an initial TC concentration of 20 mg/L, a catalyst dosage of 1 g/L, and an irradiation time of 120 min for each cycle. The reusability of the ZnO/CoFe_2_O_4_ nanocomposite was evaluated over five consecutive photocatalytic runs. After each experiment, the catalyst was recovered by centrifugation to minimize catalyst loss and ensure consistent recovery during the recycling tests, thoroughly washed with absolute ethanol and deionized water to remove adsorbed pollutants and reaction intermediates, and then vacuum-dried at 80 °C for 3 h before the subsequent run. The degradation efficiencies obtained in successive cycles are presented in Figure 8a. The photocatalyst retained high photocatalytic activity, maintaining a TC removal efficiency of 88.3% after five cycles compared with 96.8% in the first run. The slight decrease of 8.5 percentage points in degradation efficiency may be attributed to the accumulation of intermediate products, which compete with TC molecules for reactive oxygen species (ROS), as well as the partial blockage of active sites and pores by residual pollutants and degradation by-products. In addition, the complete removal of adsorbed species from the catalyst surface during the regeneration process is difficult to achieve. Nevertheless, the ZnO/CoFe_2_O_4_ nanocomposite retained high photocatalytic activity throughout repeated use, suggesting good operational stability and reusability for TC degradation [45,54]. However, because post-reaction FT-IR characterization of the recovered catalyst was not available, the structural stability of the material after photocatalysis could not be directly confirmed in the present study.

To further clarify the photocatalytic mechanism, scavenger tests were conducted to determine the contribution of the main reactive species involved in TC degradation. IPA, EDTA, BQ, and AgNO_3_ were used as specific scavengers for ·OH, h^+^, ·O_2_^−^, and e^−^, respectively. As presented in Figure 8b, the ZnO/CoFe_2_O_4_ nanocomposite achieved a TC removal efficiency of 96.8% without the addition of any scavenger, indicating the effective generation of reactive species during photocatalysis. The addition of AgNO_3_ had a negligible effect on TC degradation, with the degradation efficiency remaining relatively high at 85.6% compared with 96.8% in the absence of scavengers, suggesting that photogenerated electrons (e^−^) play only a minor role in the photocatalytic process. In contrast, the presence of BQ caused a more noticeable decrease in degradation efficiency to 79.3%, indicating that ·O_2_^−^ acts as a secondary reactive species in TC degradation. A substantial decrease in TC removal efficiency was observed in the presence of EDTA and IPA, with efficiencies dropping to 57.4% and 48.6%, respectively. The stronger inhibition caused by IPA suggests that ·OH radicals are the primary reactive species, while h^+^ also makes a significant contribution to the photocatalytic degradation process. These findings indicate that ·OH and h^+^ are the major active species responsible for TC degradation over the ZnO/CoFe_2_O_4_ photocatalyst.

To further clarify the photocatalytic mechanism beyond the scavenger results, the conduction band edge potential ($E_{CB}$) and valence band edge potential ($E_{VB}$) of ZnO and CoFe_2_O_4_ were estimated using Mulliken electronegativity theory, based on the following empirical equations [67]:

(1) $$E_{VB}=\chi -E_{e}+{0.5E}_{g}$$

(2) $$E_{CB}=E_{VB}-E_{g}$$

where χ represents the Mulliken absolute electronegativity of the semiconductor, with values of 5.79 eV for ZnO and 5.81 eV for CoFe_2_O_4_; $E_{e}$ is the energy of free electrons on the hydrogen scale, approximately 4.5 eV relative to the Normal Hydrogen Electrode (NHE); and $E_{g}$ denotes the optical bandgap energy of the photocatalyst. Based on the calculated values, the $E_{CB}$ and $E_{VB}$ of ZnO were estimated to be −0.325 and +2.905 V vs. NHE, respectively, whereas those of CoFe_2_O_4_ were +0.230 and +2.390 V vs. NHE, respectively. These estimated band-edge potentials provide a basis for assessing the thermodynamic feasibility of reactive oxygen species (ROS) generation. In particular, the $E_{VB}$ of ZnO (+2.905 V vs. NHE) is more positive than the reported redox potentials of OH^−^/·OH (+1.99 V vs. NHE) and H_2_O/·OH (+2.40 V vs. NHE), suggesting that the ZnO valence band is thermodynamically capable of oxidizing both OH^−^ and H_2_O to generate hydroxyl radicals (·OH). Meanwhile, the valence-band potential of CoFe_2_O_4_ (+2.390 V vs. NHE) is more positive than the OH^−^/·OH potential but slightly less positive than the H_2_O/·OH potential. Therefore, the CoFe_2_O_4_ valence band may support the oxidation of OH^−^ to ·OH and the direct oxidation of TC by photogenerated holes, whereas direct oxidation of H_2_O to ·OH is thermodynamically marginal. In contrast, the most negative conduction-band potential in the system (−0.325 V vs. NHE for ZnO) is only slightly less negative than the O_2_/·O_2_^−^ potential (−0.33 V vs. NHE). Therefore, direct reduction of dissolved oxygen to ·O_2_^−^ is not strongly favored based solely on the estimated band positions, although it cannot be completely excluded because the potential difference is very small and the calculated band-edge values are approximate. These results are consistent with the scavenger experiments, which suggest that ·OH and h^+^ are the dominant reactive species, whereas ·O_2_^−^ and electrons make smaller contributions to TC degradation.

Figure S5 illustrates the proposed photocatalytic degradation mechanism of TC over the ZnO/CoFe_2_O_4_ nanocomposite under visible-light irradiation. Upon light excitation, both ZnO and CoFe_2_O_4_ generate electron–hole pairs. Owing to the staggered band alignment, photogenerated electrons may migrate from the conduction band (CB) of ZnO to the CB of CoFe_2_O_4_, while holes transfer from the valence band (VB) of CoFe_2_O_4_ to the more positive VB of ZnO. This charge-transfer pathway may facilitate charge separation and reduce electron–hole recombination. The holes accumulated on the VB of ZnO possess sufficient oxidation potential to directly oxidize TC or react with H_2_O/OH^−^ to generate highly reactive hydroxyl radicals (·OH). Meanwhile, the electrons accumulated on the CB of CoFe_2_O_4_ may participate in reduction reactions involving dissolved oxygen. Therefore, h^+^ and ·OH are considered the dominant reactive species responsible for TC degradation, leading to the formation of CO_2_, H_2_O, and other degradation products. A similar mechanism has also been proposed in previous studies [68,69].

#### 2.6.4. Comparison with Previously Reported CoFe_2_O_4_-Containing Photocatalysts

The performance of the present green-synthesized ZnO/CoFe_2_O_4_ nanocomposite was compared with that of previously reported CoFe_2_O_4_-based photocatalysts prepared by different methods [45,70,71,72,73,74,75], as summarized in Table S3. The nanocomposite achieved 96.8% TC degradation at an initial concentration of 20 mg/L after 120 min of visible-light irradiation using a 50 W LED source. Accordingly, this comparison is intended to place the present system in context by highlighting the feasibility of combining a green synthesis route with a simple, relatively low-power LED irradiation system, rather than to establish its superiority. Direct comparison among the reported catalysts remains difficult because the studies differ in photocatalyst composition, pollutant concentration, solution pH, catalyst dosage, light source, and irradiation time, while some systems also employed auxiliary oxidants such as peroxymonosulfate (PMS) [71] or H_2_O_2_ [73].

## 3. Materials and Methods

### 3.1. Materials and Synthesis

Reagent specifications, lime-juice preparation, and detailed synthesis procedures are provided in Section S1 of the Supplementary Materials.

### 3.2. Characterization of Materials

The samples were characterized by XRD, FTIR, FESEM, TEM–EDX, UV–Vis DRS, XPS, nitrogen adsorption–desorption, and VSM analyses. Detailed instrumental conditions are provided in Section S2 of the Supplementary Materials.

### 3.3. Photocatalytic Experiments

Photocatalytic experiments were conducted using 50 mL of TC solution and 0.050 g of photocatalyst under irradiation from a 50 W white LED lamp (Philips, Eindhoven, The Netherlands, 6500 K) positioned 15 cm above the solution. Before irradiation, the suspension was stirred in the dark for 60 min. Aliquots were collected at 30 min intervals, and the residual TC concentration was determined by UV–Vis spectrophotometer (GENESYS 10S, Thermo Fisher Scientific, Waltham, MA, USA). The effects of solution pH, initial TC concentration, and photocatalyst dosage were investigated. Detailed procedures for the photocatalytic measurements, kinetic calculations, scavenger experiments, and reusability tests are provided in Section S3 of the Supplementary Materials. All experiments were performed in triplicate.

## 4. Conclusions

In this study, CoFe_2_O_4_ nanoparticles were synthesized using lime juice as a natural stabilizing agent and subsequently combined with ZnO to prepare a ZnO/CoFe_2_O_4_ nanocomposite. Structural and physicochemical analyses confirmed the coexistence of ZnO and CoFe_2_O_4_ without detectable secondary phases. The nanocomposite consisted predominantly of spherical and irregularly shaped nanoparticles and contained Zn, Co, Fe, and O in their expected chemical states. The composite exhibited ferromagnetic behavior, with lower saturation magnetization and higher remanence and coercivity than pure CoFe_2_O_4_. Its magnetic responsiveness suggests potential separation using an external magnetic field.

The incorporation of CoFe_2_O_4_ broadened the optical absorption of ZnO and reduced its band-gap energy from 3.23 to 3.05 eV, whereas the specific surface area and pore volume decreased relative to pure CoFe_2_O_4_. Photocatalytic experiments demonstrated that solution pH, initial TC concentration, and catalyst dosage significantly affected TC degradation. Under the optimized conditions of pH 6, an initial TC concentration of 20 mg L^−1^, and a catalyst dosage of 1 g L^−1^, the nanocomposite achieved 96.8% TC degradation after 120 min of visible-light irradiation, with a pseudo-first-order rate constant of 0.029 min^−1^. The degradation efficiency remained 88.3% after five consecutive cycles. Reactive-species trapping experiments indicated that photogenerated holes (h^+^) and hydroxyl radicals (·OH) were the dominant species involved in TC degradation. The improved photocatalytic activity may be associated with interfacial interactions between ZnO and CoFe_2_O_4_, suggesting that the green-synthesized nanocomposite has potential as a visible-light photocatalyst for TC degradation under the investigated conditions.

## Figures and Tables

**Table 1:** The weight percentage (wt.%) and atomic percentage (at.%) of the O, Fe, Zn, and Co elements for the samples (**a**) ZnO, (**b**) CoFe_2_O_4_, and (**c**) ZnO/CoFe_2_O_4_.

Element	Weight Percentage (Theoretical)	Weight Percentage (Experimental)	Atomic Percentage (Theoretical)	Atomic Percentage (Experimental)
(**a**) ZnO				
O K	19.66	20.95	50	51.99
Zn K	80.34	79.05	50	48.01
(**b**) CoFe_2_O_4_				
O K	27.27	26.16	57.14	55.72
Fe K	47.60	49.20	28.57	30.03
Co K	25.13	24.64	14.29	14.25
(**c**) ZnO/CoFe_2_O_4_				
O K	22.44	21.89	52.94	52.3
Fe K	17.40	14.92	11.76	10.21
Co K	9.18	8.67	5.88	5.62
Zn K	50.98	54.52	29.42	31.87

## Data Availability

The original contributions presented in the study are included in the article. Further inquiries can be directed to the corresponding author.

## Supplementary Materials

The following supporting information can be downloaded at: https://www.mdpi.com/article/10.3390/molecules31162772/s1, Section S1: Reagents and Detailed Synthesis Procedures; Section S2: Detailed Characterization Procedures; Section S3: Photocatalytic Measurements; Section S4: Crystallographic Parameter Calculations; Figure S1: FE-SEM and TEM images of the pristine materials: (a,b) ZnO NPs and (c,d) CoFe_2_O_4_ NPs; Figure S2: EDX spectra of (a) ZnO NPs, (b) CoFe_2_O_4_ NPs, and (c) ZnO/CoFe_2_O_4_ nanocomposite; Figure S3: N_2_ adsorption–desorption isotherms and corresponding BJH pore-size distributions of (a) ZnO and (b) CoFe_2_O_4_; Figure S4: Determination of the point of zero charge (${pH}_{PZC}$) of the ZnO/CoFe_2_O_4_ nanocomposite; Figure S5: Proposed photocatalytic degradation pathway of TC over the ZnO/CoFe_2_O_4_ nanocomposite under visible-light irradiation; Figure S6: Schematic diagram of the photocatalytic reactor system; Table S1: Crystallite size (D), lattice parameters (a, b, and c), unit-cell volume (V), and X-ray density (***ρ***) of ZnO, CoFe_2_O_4_, and ZnO/CoFe_2_O_4_; Table S2: BET surface area, pore volume, and pore size of ZnO, CoFe_2_O_4_, and ZnO/CoFe_2_O_4_. Table S3: Summary of reported CoFe_2_O_4_-based photocatalysts for tetracycline degradation. References [25,26,27,45,70,71,72,73,74,75,76] are cited in the Supplementary Materials.
