# Supplementary material for: Green Synthesis and Characterization of ZnO/CoFe2O4 Nanocomposites for Photocatalytic Degradation of Tetracycline Under Visible Light
Source: Molecules. 2026 Aug 9;31(16):2772. doi: 10.3390/molecules31162772 (PMC13515848; doi:10.3390/molecules31162772)
Supplement: Supplementary file 1 [file molecules-31-02772-s001.zip › molecules-4364839-supplementary.pdf]

molecules

MDPI

Article

Green Synthesis and Characterization of $ZnO/CoFe_2O_4$ Nanocomposites for Photocatalytic Degradation of Tetracycline under Visible Light

Phan Thi Minh Huyen and Nguyen Xuan Dung *

College of Education, Vinh University, Vinh City 43131, Vietnam; huyenptm@vinhuni.edu.vn
* Correspondence: dungnx@vinhuni.edu.vn; Tel.: +84-912-918-313

Table of contents

S1. Reagents and Detailed Synthesis Procedures 1
S2. Detailed Characterization Procedures 2
S3. Photocatalytic Measurements 2
S4. Crystallographic Parameter Calculations 4

S1. Reagents and Detailed Synthesis Procedures

S1.1. Reagents

All chemicals were of analytical grade and used without further purification. Zinc acetate dihydrate ($Zn(CH_3COO)_2{\cdot}2H_2O$, 99%), cobalt(II) nitrate hexahydrate ($Co(NO_3)_2{\cdot}6H_2O$, 99%), iron(III) nitrate nonahydrate ($Fe(NO_3)_3{\cdot}9H_2O$, 99%), tetracycline (TC, 98%), sodium hydroxide (NaOH, 97%), p-benzoquinone (BQ, $C_6H_4O_2$, 98%), isopropanol (IPA, C_3H_8O, 99.5%), disodium ethylenediaminetetraacetate dihydrate ($Na_2EDTA{\cdot}2H_2O$, 99%), silver nitrate ($AgNO_3$, 99%), and absolute ethanol (C_2H_5OH, 99.8%) were supplied by Sigma-Aldrich (St. Louis, MO, USA). Acetic acid (CH_3COOH, 99%), diethylene glycol (DEG, $C_4H_{10}O_3$, 99%), sodium nitrate ($NaNO_3$, 99%), nitric acid (HNO_3, 65–68%), and hydrochloric acid (HCl, 37%, density = 1.19 g mL^{-1}) were obtained from Aladdin Reagent Co., Ltd. (Shanghai, China). Deionized water was used throughout the experiments.

S1.2. Preparation of Lime Juice

Fresh green limes obtained from a supermarket in Nghe An Province, Vietnam, were thoroughly washed with deionized water. The juice was manually extracted, centrifuged at 13,000 rpm for 10 min, and filtered to remove suspended solids. The clarified lime juice was stored at 4 °C until use.

https://doi.org/10.3390/xxxxx

S1.3. *Synthesis of Materials*

$CoFe_2O_4$ nanoparticles were prepared by dissolving 1.455 g of $Co(NO_3)_2·6H_2O$ and 4.040 g of $Fe(NO_3)_3·9H_2O$ in 50 mL of deionized water under continuous stirring. Clarified lime juice (20 mL) was added at room temperature, and the mixture was stirred at 70–80 °C until gel formation. The gel was dried at 80 °C for 3 h, heated to 500 °C at 5 °C min^{-1}, calcined at 500 °C for 1 h in air, and allowed to cool naturally to room temperature. ZnO nanoparticles were prepared using the same lime-juice-assisted procedure, except that 3.293 g of $Zn(CH_3COO)_2·2H_2O$ was used as the metal precursor.

For the ZnO/$CoFe_2O_4$ nanocomposite, 0.469 g of the synthesized $CoFe_2O_4$ was dispersed in 30 mL of DEG and ultrasonicated for 20 min. A solution of 2.195 g of $Zn(CH_3COO)_2·2H_2O$ in 50 mL of deionized water was gradually added under continuous stirring, followed by 20 mL of clarified lime juice. The mixture was stirred at 70–80 °C for approximately 3 h, dried, and calcined at 500 °C to obtain the ZnO/$CoFe_2O_4$ nanocomposite.

S2. Detailed Characterization Procedures

The crystalline phases and structural properties were examined by X-ray diffraction (XRD) using a D8 Advance diffractometer (Bruker, Karlsruhe, Germany) equipped with Cu Kα radiation (λ = 0.15406 nm). Diffraction patterns were recorded over a 2θ range of 10–70°.

Surface functional groups and possible interactions between ZnO and $CoFe_2O_4$ were investigated by Fourier-transform infrared spectroscopy (FTIR) using a Thermo-Nicolet Nexus 670 instrument (Thermo Fisher Scientific, Madison, WI, USA) and the KBr pellet method over the range of 4000–400 cm^{-1}.

Surface morphology and particle size were examined by scanning electron microscopy (SEM, S-4800, Hitachi High-Tech Corporation, Tokyo, Japan) and transmission electron microscopy (TEM, JEM-1400, JEOL Ltd., Tokyo, Japan). Elemental composition was analyzed by energy-dispersive X-ray spectroscopy (EDX, 7593-H, HORIBA, Ltd., Kyoto, Japan) integrated with the SEM system.

The optical properties of the solid samples were evaluated by UV–Vis diffuse reflectance spectroscopy (UV–Vis DRS) using a Cary 5000 UV–Vis–NIR spectrometer (Agilent Technologies, Santa Clara, CA, USA) with $BaSO_4$ as the white reflectance reference. Surface elemental composition and chemical states were examined by X-ray photoelectron spectroscopy (XPS) using an ESCALab 250 spectrometer (Thermo VG Scientific, East Grinstead, UK).

Textural properties, including specific surface area and pore characteristics, were evaluated from nitrogen adsorption–desorption isotherms using the Brunauer–Emmett–Teller (BET) method on a Tristar-3000 analyzer (Micromeritics, Norcross, GA, USA).

The magnetic properties of the samples were measured using a vibrating sample magnetometer (VSM, 7404, Lake Shore Cryotronics, Inc., Westerville, OH, USA).

S3. Photocatalytic Measurements

S3.1. *Standard Photocatalytic Procedure, Removal Efficiency, and Kinetic Analysis*

Unless otherwise stated, photocatalytic experiments were conducted at room temperature in an open glass vessel containing 50 mL of TC solution (20 mg L^{-1}) and 0.050 g of photocatalyst, corresponding to a catalyst dosage of 1.0 g L^{-1}, at pH 6. Before irradiation, the suspension was stirred in the dark for 60 min to establish adsorption–desorption equilibrium. The suspension was then irradiated for 120 min using a 50 W white LED lamp (Philips, Eindhoven, Netherlands, 6500 K) positioned 15 cm above the solution surface

while being continuously stirred. The reactor remained open to ambient air, and no external oxidant was added. Aliquots were withdrawn at 30 min intervals and analyzed using a UV–Vis spectrophotometer (GENESYS 10S, Thermo Fisher Scientific, Waltham, MA, USA) to determine the residual TC concentration. A schematic diagram of the reactor is shown in Figure S6. All photocatalytic experiments were performed in triplicate.

$$RE\ (\%) = \frac{C_0 - C_t}{C_0} \times 100\% \quad \text{(S1)}$$

where C_t (mg/L) and C_o (mg/L) correspond to the concentration of TC at time t and the initial concentration, respectively. The degradation kinetics were evaluated using the pseudo-first-order model:

$$\ln\frac{C_0}{C_t} = kt \quad \text{(S2)}$$

where k is the pseudo-first-order rate constant (min^{-1}), and t is the irradiation time (min). The k value was obtained from the slope of the linear plot of $\ln(C_0/C_t)$ versus t.

S3.2. Effects of Operating Parameters

The effect of initial solution pH was investigated at pH 2, 4, 6, 8, and 10 using an initial TC concentration of 20 mg/L and a catalyst dosage of 1.0 g/L. The solution pH was adjusted using dilute HCl or NaOH solutions.

The effect of initial TC concentration was evaluated at 5, 10, 20, 30, and 40 mg/L at pH 6 using a catalyst dosage of 1.0 g/L. The photocatalyst dosage was varied from 0.5 to 2.0 g/L using 20 mg/L TC at pH 6. All other conditions were maintained as described in Section S3.1.

S3.3. Determination of the Point of Zero Charge

The point of zero charge (pH_{PZC}) of the $ZnO/CoFe_2O_4$ nanocomposite was determined using the pH-drift method [1]. A series of 0.1 M $NaNO_3$ solutions (50 mL each) was prepared, and the initial pH values (pH_i) were adjusted from 3 to 10 using dilute HNO_3 or NaOH solutions. Subsequently, 0.1 g of the photocatalyst was added to each solution, and the suspensions were continuously stirred at room temperature for 48 h to attain equilibrium. The final pH values (pH_f) were then measured, and the differences between the final and initial pH values were calculated as $\Delta pH = pH_f - pH_i$. The pH_{PZC} was determined from the initial pH at which ΔpH was equal to zero.

S3.4. Reactive-Species Scavenging Experiments

Reactive-species scavenging experiments were performed to identify the principal active species involved in TC degradation. For each 50 mL reaction solution, 27.0 mg of p-benzoquinone (BQ; 5.0 mM), approximately 19 μL of isopropanol (IPA; approximately 5.0 mM), 93.1 mg of $Na_2EDTA·2H_2O$ (5.0 mM), or 4.25 mg of $AgNO_3$ (0.50 mM) was separately added as a scavenger for $·O_2^-$, ·OH, h^+, or e^-, respectively.

All scavenging experiments were conducted under the standard conditions described in Section S3.1. Each experiment was performed in triplicate, and the results are reported as mean values.

S3.5. Reusability and Operational Stability Tests

The reusability and operational stability of the $ZnO/CoFe_2O_4$ nanocomposite were evaluated over five consecutive photocatalytic cycles. After each cycle, the photocatalyst was recovered by centrifugation, washed thoroughly with absolute ethanol and deionized water to remove residual TC and degradation products, vacuum-dried at 80 °C for 3 h, and reused in fresh TC solution under the same experimental conditions.

The photocatalytic removal efficiency obtained in each cycle was used to assess the reusability and operational stability of the $ZnO/CoFe_2O_4$ nanocomposite. All recycling experiments were performed in triplicate, and the results are presented as mean values.

S4. Crystallographic Parameter Calculations

The average crystallite size, D, was calculated using the Scherrer equation [2]:

$$D = \frac{K\lambda}{\beta\cos\theta} \quad \text{(S3)}$$

where K is the shape factor (K = 0.9), λ is the wavelength of Cu Kα radiation (1.5406 Å), β is the full width at half maximum (FWHM) of the selected diffraction peak, and θ is the corresponding Bragg angle. The (101) reflection of ZnO and the (311) reflection of $CoFe_2O_4$ were used for the calculations.

For hexagonal ZnO, the lattice constants (a and c), unit-cell volume (V), and X-ray density (ϱ) were calculated from the (100) and (002) reflections using Equations (S4)–(S7) [3]:

$$a = \sqrt{\frac{4d_{100}^2}{3}} \quad \text{(S4)}$$

$$c = 2d_{002} \quad \text{(S5)}$$

$$V = \frac{\sqrt{3}}{2}a^2c \quad \text{(S6)}$$

$$\rho = \frac{nM}{NV} \quad \text{(S7)}$$

where M is the molar mass of ZnO (81.38 g mol^{-1}), n = 2 is the number of formula units per unit cell, and N is Avogadro's constant (6.022 × 10^{23} mol^{-1}).

For cubic $CoFe_2O_4$, the corresponding crystallographic parameters were calculated using Equations (S8)–(S10) [4]:

$$a = d_{hkl}\sqrt{(h^2 + k^2 + l^2)} \quad \text{(S8)}$$

$$V = a^3 \quad \text{(S9)}$$

$$\rho = \frac{8M}{NV} \quad \text{(S10)}$$

where M is the molar mass of $CoFe_2O_4$, and N is Avogadro's constant.

The calculated crystallographic parameters are summarized in Table S1.

Table S1. Crystallite size (D), lattice parameters (a, b, and c), unit-cell volume (V), and X-ray density (ρ) of ZnO, $CoFe_2O_4$, and $ZnO/CoFe_2O_4$.

Material	*D* (nm)	*a* (Å)	*b* (Å)	*c* (Å)	*V* ($Å^3$)	ρ (g cm^{-3})
ZnO	15.90	3.2359	3.2359	5.1866	47.03	5.75
$CoFe_2O_4$	11.03	8.3326	8.3326	8.3326	578.56	5.39
ZnO in the nanocomposite	21.67	3.2408	3.2408	5.1939	47.24	5.72
$CoFe_2O_4$ in the nanocomposite	12.84	8.3666	8.3666	8.3666	585.67	5.32
Standard ZnO (JCPDS No. 36-1451)	—	3.2490	3.2490	5.2060	47.59	5.68
Standard $CoFe_2O_4$ (JCPDS No. 22-1086)	—	8.3920	8.3920	8.3920	591.01	5.27

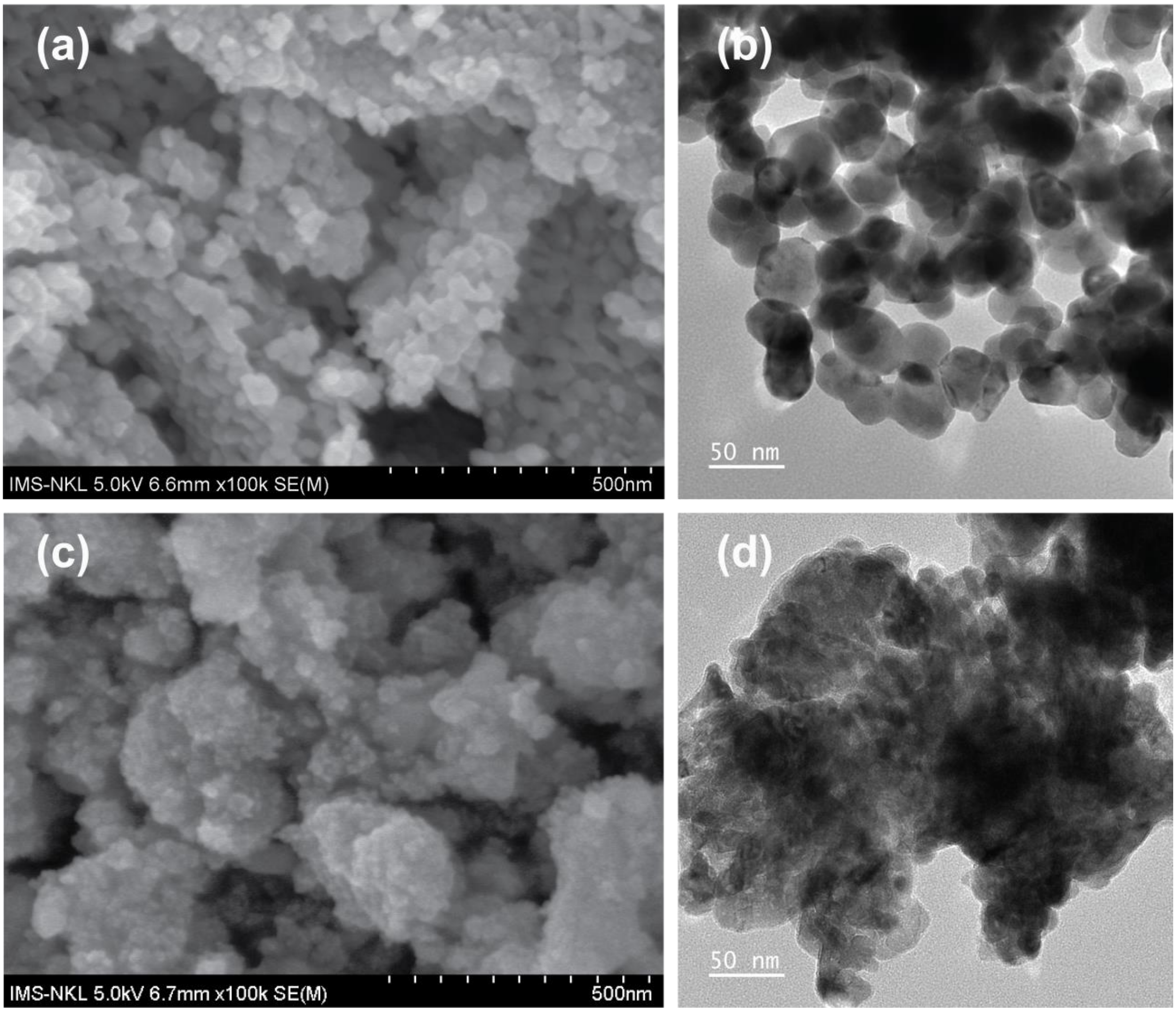

Figure S1. FE-SEM and TEM images of the pristine materials: (a,b) ZnO NPs and (c,d) $CoFe_2O_4$ NPs.

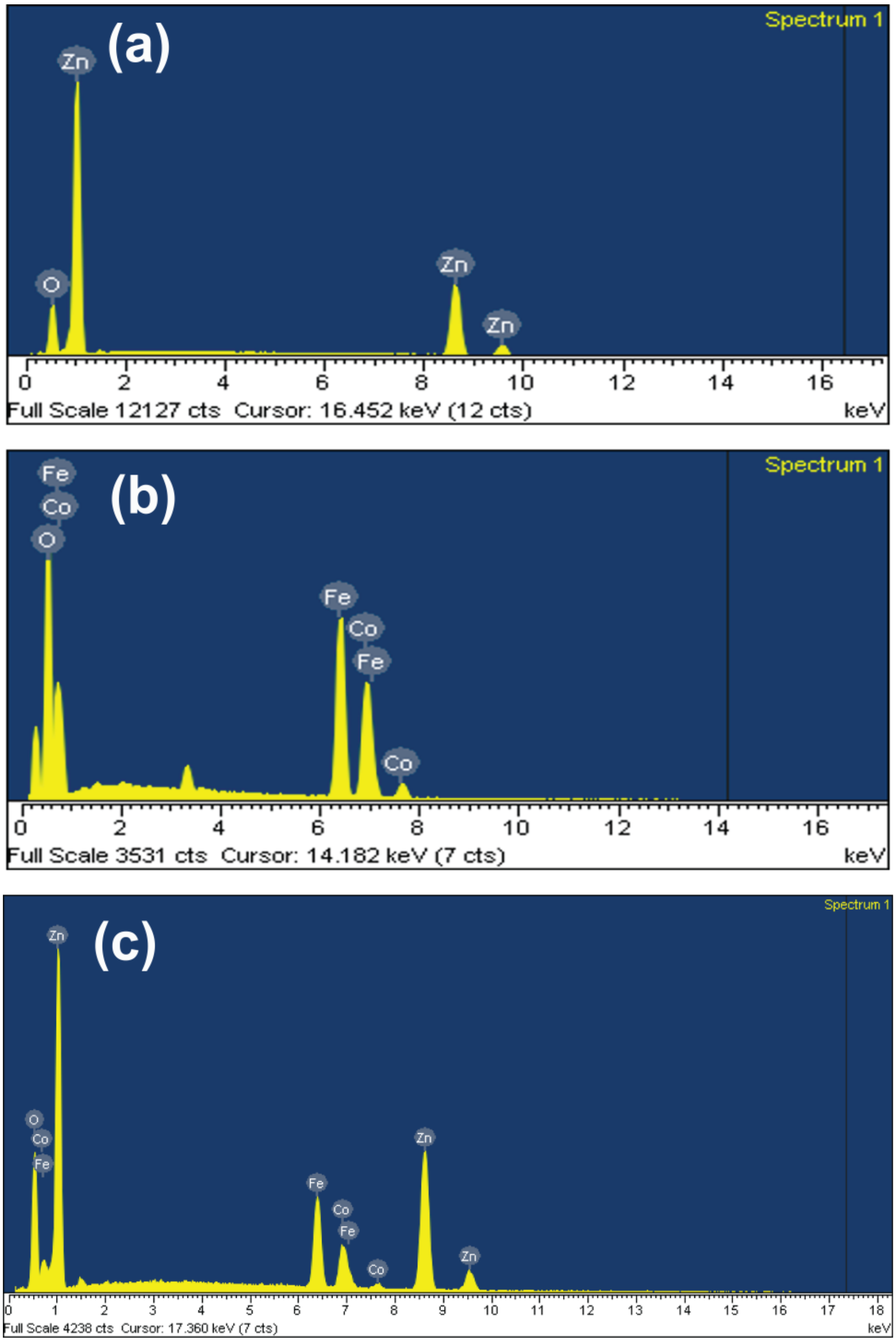

Figure S2. EDX spectra of (a) ZnO NPs, (b) $CoFe_2O_4$ NPs, and (c) ZnO/$CoFe_2O_4$ nanocomposite.

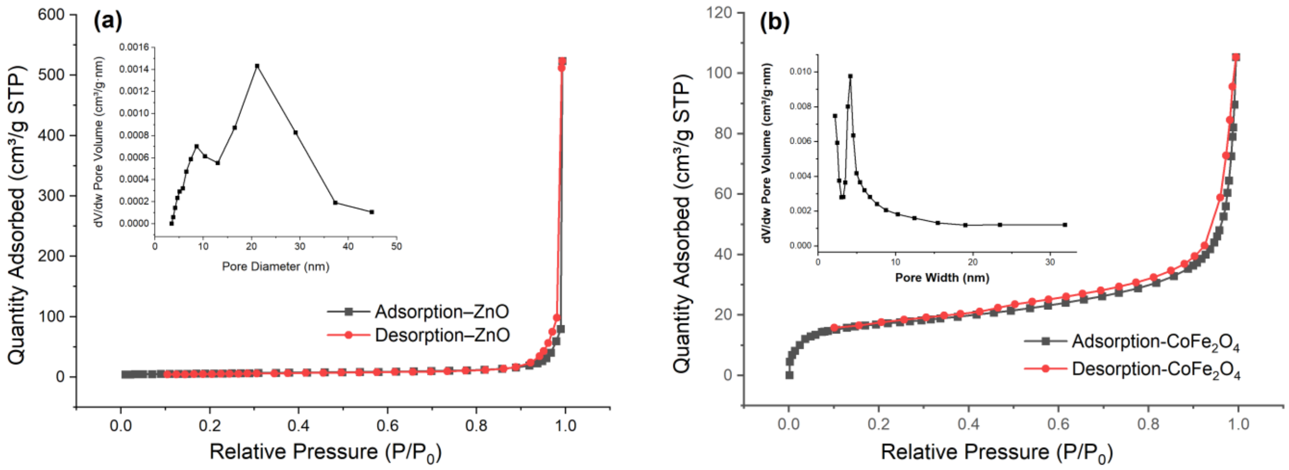

Figure S3. N_2 adsorption–desorption isotherms and corresponding BJH pore-size distributions of (a) ZnO and (b) $CoFe_2O_4$.

Table S2. BET surface area, pore volume, and pore size of ZnO, $CoFe_2O_4$, and ZnO/$CoFe_2O_4$.

Material	BET surface area ($m^2\ g^{-1}$)	Pore volume ($cm^3\ g^{-1}$)	Pore size (nm)
ZnO	10.9	0.011	15.7
$CoFe_2O_4$	75.3	0.157	11.1
ZnO/$CoFe_2O_4$	36.8	0.022	3.5

Table S3. Summary of reported $CoFe_2O_4$-based photocatalysts for tetracycline degradation.

**Photocatalyst**	**Synthesis route of $CoFe_2O_4$ NPs**	**Light source**	**TC/OTC concentration (mg/L)**	**Solution pH/ Catalyst dosage (g/L)**	**Degradation efficiency (%)**	**Time (min)**	**Ref.**
$CoFe_2O_4$/MWCNTs	Co-precipitation	Visible light (120 W lamp)	50	7/0.6	90.0	120	[5]
$CoFe_2O_4$/$NaBiO_3$	Sol–gel	Visible light (500 W Xe lamp)	10	NR/1.0	94	100	[6]
Sulfur-doped $CoFe_2O_4$	Calcination	Visible light (300 W tungsten lamp)	80 (OTC)	8.5/1.0	90.4	300	[7]
ZnO/$CoFe_2O_4$	Hydrothermal method + Calcination	Visible light (300 W Xe lamp)	20	NR/0.3	81.03	120	[8]
$CoFe_2O_4$/WO_3	Hydrothermal method + Calcination	Visible light (Xe lamp)	20	NR/0.2	77.0	20	[9]
TiO_2/$CoFe_2O_4$	Sol–gel	Visible light	10	NR/0.1	50.4	180	[10]
KPCN/GO/$CoFe_2O_4$	Hydrothermal method	Visible light (500 W Halogen lamp)	38	7/0.6	85.0	60	[11]
ZnO/$CoFe_2O_4$	Green synthesis	Visible light (50 W LED)	20	6/1.0	96.8	120	This work

Note: OTC, oxytetracycline; NR, not reported;

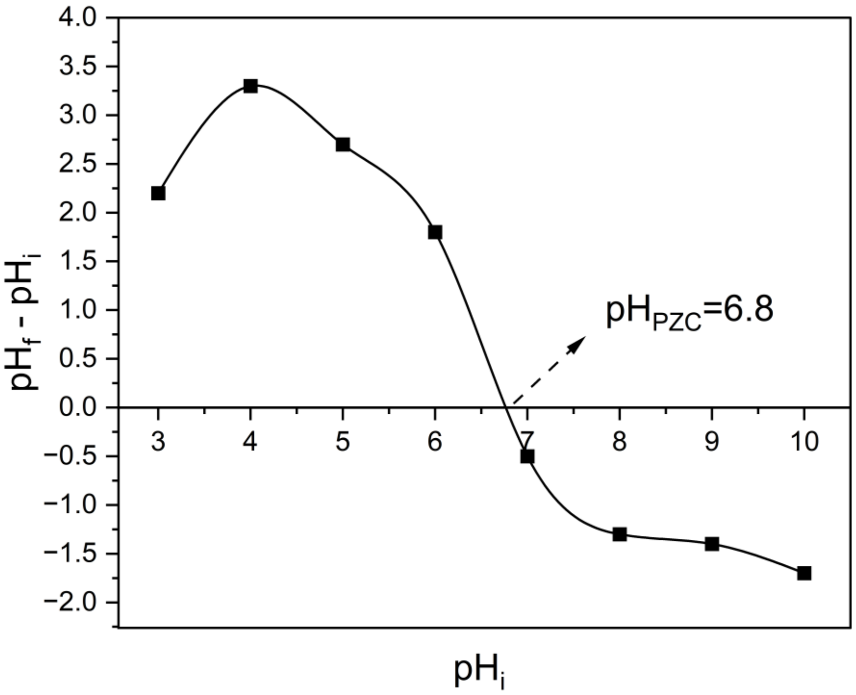

Figure S4. Determination of the point of zero charge (pH_{PZC}) of the ZnO/$CoFe_2O_4$ nanocomposite.

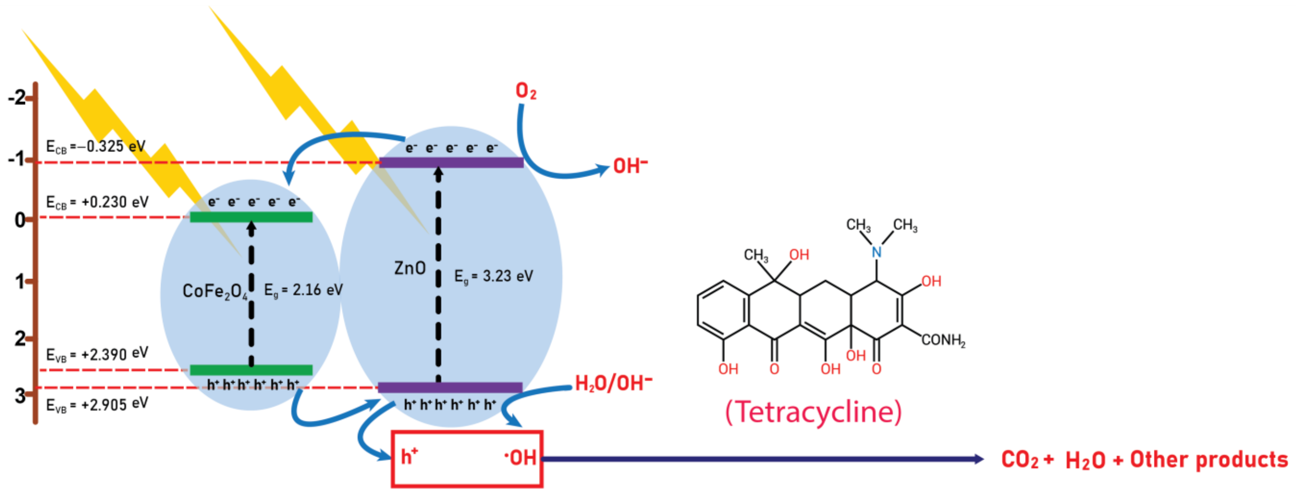

Figure S5. Proposed photocatalytic degradation pathway of TC over the ZnO/$CoFe_2O_4$ nanocomposite under visible-light irradiation.

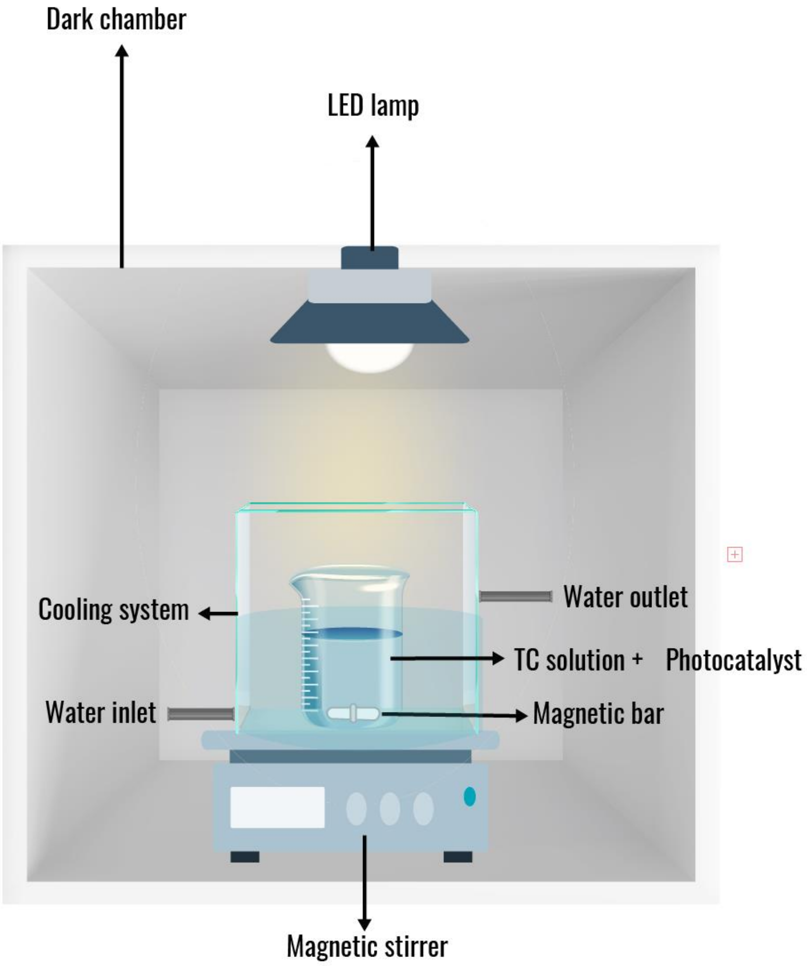

Figure S6. Schematic diagram of the photocatalytic reactor system.

References

1. Alorabi, A.Q. Juniperus Hydrochar Decorated with Chitosan/Zinc Oxide Nanocomposite for Efficient Removal of Malachite Green Dye from Water. *Journal of Saudi Chemical Society* **2025**, *29*, 42, doi:10.1007/s44442-025-00038-6.
2. Patterson, A.L. The Scherrer Formula for X-Ray Particle Size Determination. *Physical review* **1939**, *56*, 978–982, doi:10.1103/PhysRev.56.978.
3. Katiyar, A.; Kumar, N.; Srivastava, P.; Shukla, R.K.; Srivastava, A. Structural and Physical Parameters of Sol-Gel Spin Coated ZnO Thin Films: Effect of Sol Concentration. *Mater. Today Proc.* **2020**, *29*, 1098–1103, doi:10.1016/j.matpr.2020.05.167.
4. Infancy, W.T.; Vinosha, P.A.; Xavier, B.; Dinesh, A.; Patil, R.P.; Ayyar, M.; Slimani, Y.; Almessiere, M.A.; Baykal, A.; Iqbal, M. Tunable Optical, Morphological, and Magnetic Properties of $CoFe_2O_4$/MXene Nanocomposites for Reusable Photocatalytic Water Remediation. *Journal of Materials Science: Materials in Electronics* **2025**, *36*, 917, doi:10.1007/s10854-025-14884-y.
5. Varghese, D.; Michael, J.R.R.; Patrick, J.S.J.; Dominic, A.; Paul Winston, A.J.P.; Sankar, M.; Joseph, M.; Daniel, S.; Moses, V.A.R. Efficient Visible-Light-Driven Photocatalytic Degradation of Tetracycline by Rod-like $CoFe_2O_4$/MWCNTs Nanocomposite as a Recyclable Photocatalyst: A Comprehensive Study. *Int. J. Environ. Anal. Chem.* **2025**, *105*, 536–557, doi:10.1080/03067319.2023.2264787.
6. Zhang, J.; Zhang, S.; Bian, X.; Yin, Y.; Huang, W.; Liu, C.; Liang, X.; Li, F. High Efficiency Removal Performance of Tetracycline by Magnetic $CoFe_2O_4$/$NaBiO_3$ Photocatalytic Synergistic Persulfate Technology. *Molecules* **2024**, *29*, 4055, doi:10.3390/molecules29174055.

7. Li, R.; Sun, C.; Liu, J.; Zhen, Q. Sulfur-Doped $CoFe_2O_4$ Nanopowders for Enhanced Visible-Light Photocatalytic Activity and Magnetic Properties. *RSC Adv.* **2017**, *7*, 50546–50554, doi:10.1039/C7RA10016G.
8. Ye, L.; Yan, C.; Jiang, Y.; Yang, Q.; Lu, C.; Guan, W. Preparation of Hollow Cone-like $ZnO/CoFe_2O_4$ Heterostructures and Their Photocatalytic Properties. *Micro Nano Lett.* **2015**, *10*, 202–205, doi:10.1049/mnl.2014.0586.
9. Dong, S.; Dai, J.; Yang, Y.; Zada, A.; Qi, K. Extended Interfacial Charge Transference in $CoFe_2O_4/WO_3$ Nanocomposites for the Photocatalytic Degradation of Tetracycline Antibiotics. *Molecules* **2024**, *29*, 4561, doi:10.3390/molecules29194561.
10. Ibrahim, I.; Belessiotis, G. V.; Elseman, A.M.; Mohamed, M.M.; Ren, Y.; Salama, T.M.; Mohamed, M.B.I. Magnetic $TiO_2/CoFe_2O_4$ Photocatalysts for Degradation of Organic Dyes and Pharmaceuticals without Oxidants. *Nanomaterials* **2022**, *12*, 3290, doi:10.3390/nano12193290.
11. Kumar, R.; Sudhaik, A.; Sonu; Nguyen, V.-H.; Van Le, Q.; Ahamad, T.; Thakur, S.; Kumar, N.; Hussain, C.M.; Singh, P.; et al. Graphene Oxide Modified K, P Co-Doped g-C3N4 and CoFe2O4 Composite for Photocatalytic Degradation of Antibiotics. *J. Taiwan Inst. Chem. Eng.* **2023**, *150*, 105077, doi:10.1016/j.jtice.2023.105077.
